# De-escalation of biological therapy in inflammatory bowel disease patients following prior dose escalation

**DOI:** 10.1097/MEG.0000000000002336

**Published:** 2022-03-01

**Authors:** Pepijn W. A. Thomas, Lisa J. T. Smits, Maarten Te Groen, Rachel L. West, Maurice G. V. M. Russel, Jeroen M. Jansen, Tessa E. H. Römkens, Frank Hoentjen

**Affiliations:** a Department of Gastroenterology and Hepatology, Radboud University Medical Centre, Nijmegen; b Department of Gastroenterology and Hepatology, Franciscus Gasthuis & Vlietland, Rotterdam; c Department of Gastroenterology and Hepatology, Medical Spectrum Twente, Enschede; d Department of Gastroenterology and Hepatology, Onze Lieve Vrouwe Gasthuis, Amsterdam; e Department of Gastroenterology and Hepatology, Jeroen Bosch Hospital, 's-Hertogenbosch, The Netherlands; f Division of Gastroenterology, Department of Medicine, University of Alberta, Edmonton, Canada

**Keywords:** adalimumab, biological, Crohn’s disease, de-escalation, inflammatory bowel disease, infliximab, ulcerative colitis, vedolizumab

## Abstract

**Background:**

Limited data are available on biological therapy de-escalation after prior escalation in inflammatory bowel disease (IBD) patients. This study aimed to assess the frequency and success rate of de-escalation of biological therapy in IBD patients after prior dose escalation and to evaluate which measures are used to guide de-escalation.

**Methods:**

This multicentre retrospective cohort study enrolled IBD patients treated with infliximab (IFX), adalimumab (ADA) or vedolizumab (VEDO) in whom therapy was de-escalated after prior biological escalation. De-escalations were considered pharmacokinetic-driven if based on clinical symptoms combined with therapeutic or supratherapeutic trough levels, and disease activity-driven if based on faecal calprotectin less than or equal to 200 µg/g or resolution of perianal fistula drainage or closure or endoscopic remission. Successful de-escalation was defined as remaining on the same or lower biological dose for greater than or equal to 6 months after de-escalation without the need for corticosteroids.

**Results:**

In total, 206 IFX users, 85 ADA users and 55 VEDO users underwent therapy escalation. Of these patients, 34 (17%) on IFX, 18 (21%) on ADA and 8 (15%) on VEDO underwent therapy de-escalation. De-escalation was successful in 88% of IFX patients, 89% of ADA and 100% of VEDO. The probability of remaining on the de-escalated regimen or further de-escalation after 1 year was 85% for IFX, 62% for ADA and 100% for VEDO. Disease activity-driven de-escalations were more often successful (97%) than pharmacokinetic- and no marker-driven de-escalations (76%); *P* = 0.017.

**Conclusion:**

De-escalation after biological dose escalation was successful in the majority of carefully selected IBD patients. Objective assessment of remission increased the likelihood of successful de-escalation.

## Introduction

Biologicals are an effective treatment to achieve induction and maintenance of remission in inflammatory bowel disease (IBD). However, up to 46% of patients lose response over time while dose escalation may be attempted to regain effect [1]. Dose escalation rates vary between 23 and 56% [2–4] and restores responsiveness in more than half of the patients [2,3,5]. However, it is unclear whether these patients may only need an escalated dose for a brief period or alternatively, require sustained dose escalation. Potential overtreatment results in high costs, frequent clinical visits and a potentially higher risk of serious infections and other adverse events [6–8].

To overcome these concerns, treatment may be de-escalated or even discontinued. However, discontinuation is associated with a high relapse risk. Therefore, both physicians and patients are generally reluctant to select this option [9,10]. Dose de-escalation is an alternative strategy to maintain remission while using the lowest possible dose. Generally, objective markers of disease activity are warranted before dose escalation or de-escalation to avoid symptom-based therapeutic changes.

Despite a lack of clear recommendations for de-escalation, several studies have reported dose de-escalation rates that varied between 27 and 60% [2,3,11,12]. However, few studies have assessed the success rate of dose de-escalation after previous dose escalation. Two retrospective studies showed success rates of 63 and 80% after de-escalation of adalimumab (ADA) [2,3]. However, these studies were performed before the wide implementation of objective measures of inflammation in regular IBD care. A more recent retrospective cohort study showed that de-escalation of infliximab (IFX) was successful in 75% of patients with preceding biochemical remission [13]. There is still sparse data on de-escalation outcomes and which measures are used in decision-making before de-escalation. Therefore, this study aimed to assess (1) the frequency and outcomes of de-escalation of biological therapy in IBD patients after prior dose escalation and (2) which disease measures are used to initiate de-escalation.

## Methods

### Study design

This retrospective multicentre cohort study assessed the outcome of de-escalation of biological therapy in IBD patients following prior dose escalation, and the proportion of de-escalation based on objective markers of disease activity, using the IBDREAM registry. IBDREAM systematically records prospective data from IBD patients in four nonacademic hospitals and one academic hospital in the Netherlands, as described previously [14–16].

### Study population

Patients aged greater than or equal to 16 years with an established diagnosis of Crohn’s disease, ulcerative colitis or IBD-unclassified were eligible if they initiated biological therapy [IFX, ADA and vedolizumab (VEDO)] after January 2013 and had at least one biological escalation and subsequently one de-escalation. Participants were excluded if they had not received the regular induction dose (i.e. 5 mg/kg at 0, 2 and 6 weeks for IFX; 160–80 mg at week 0 and 2, followed by 40 mg every other week for ADA; and 300 mg at 0, 2 and 6 weeks for VEDO), were primary nonresponder, used only one dose of the escalated regimen, used only one dose of the de-escalated regimen or follow-up after de-escalation was less than 6 months. Only the first de-escalation of the biological treatment was analysed in more detail in this study.

### Data collection

For this study, we extracted data on demographics, disease location and behaviour according to the Montreal classification, previous and concomitant IBD medication use, reasons for discontinuation, faecal calprotectin levels and endoscopic assessment within 3 months before de-escalation, medication trough levels within 6 months before de-escalation, endoscopic procedures, IBD-related surgery and IBD-related hospital admissions. Data were extracted from the IBDREAM registry on 6 June 2020.

### Outcomes and definitions

#### Primary outcome

The primary outcome was the proportion of IBD patients with a successful de-escalation after a previous successful escalation. Successful de-escalation was defined as remaining on the same or lower biological dose for greater than or equal to 6 months after de-escalation without the need for corticosteroids [2,3]. De-escalation was defined as a dose reduction of the previously escalated dose of at least 2.5 mg/kg for IFX to either 7.5 or 5 mg/kg, or interval increase of at least one week for IFX or VEDO to once every 5–8 weeks, or ADA to once every 2 weeks. Escalation was defined as a dose increase of at least 2.5 mg/kg for IFX to either 7.5 or 10 mg/kg, or interval reduction of at least one week for IFX or VEDO to once every 4–7 weeks, or ADA to once every week. These cutoffs were selected to capture only clinically relevant dose escalations and dose de-escalations and to exclude dosing corrections based on, for example, weight changes [13].

#### Secondary outcomes

Secondary outcomes included characteristics of de-escalation, drug survival after de-escalation, de-escalation in time frames 2013–2016 and 2017–2019, the proportion of de-escalations that was performed based on diagnostic measures and outcomes stratified for a de-escalation reason. Characteristics included the time on the preceding escalated dose, change in dose and dosing interval, trough levels and reasons for unsuccessful de-escalation. Drug survival after de-escalation was defined as the time continuing the de-escalated regimen or further de-escalation until an intervention occurred including surgery, dose escalation, corticosteroid use, discontinuation due to adverse events or loss of response. Patients were censored if they had not discontinued the drug at the time of data collection or were lost to follow-up. Time frames were used to assess differences in de-escalation patterns over time, as the use of objective disease markers for decision-making has been more widely applied in clinical practice in the more recent years.

#### Disease activity-driven, pharmacokinetic-driven and no marker-driven de-escalations

De-escalations were considered disease activity-driven if based on faecal calprotectin less than or equal to 200 µg/g [17], resolution of perianal fistula drainage or closure or endoscopic remission based on the absence of ulcers. De-escalations were considered pharmacokinetic-driven if they were based on therapeutic or supratherapeutic trough levels combined with clinical remission, in the absence of criteria for disease activity. Therapeutic trough levels were defined as IFX greater than or equal to 3 µg/ml, ADA greater than 5.3 µg/ml or VEDO greater than 14 µg/ml and supratherapeutic trough levels as IFX greater than 7 µg/ml according to previously published standards [18–20]. De-escalations were considered no marker-driven if this was based on only the absence of clinical symptoms or if diagnostic measures did not fulfil the criteria for pharmacokinetic-driven or disease activity-driven.

### Statistical analysis

Non-normally distributed continuous variables were presented as median with interquartile range (IQR) and compared by using the Mann–Whitney *U* test. Normally distributed continuous variables were presented as mean with SD. Categorical variables were presented as number (*N*) and percentages and were compared by using the chi-square test. The Kaplan–Meier curve was used to present the drug survival after de-escalation. To assess the impact of the use of objective disease measures (faecal calprotectin and endoscopic assessment) on de-escalation outcomes, we compared two pooled groups: (1) de-escalation based on objective disease measures including disease activity-driven and a combination of disease activity-driven and pharmacokinetic-driven de-escalations and (2) de-escalations not based on objective disease measures including no marker-driven and pharmacokinetic-driven only de-escalations. The Fisher’s exact test was used to compare outcomes of de-escalations based on objective disease measures and de-escalations not based on objective disease measures. A *P* value less than 0.05 was considered statistically significant. SPSS Statistics (version 26.0; IBM Corp, Armonk, New York, USA) was used for statistical analyses.

### Ethical approval

This study was reviewed and approved by the Radboudumc Committee on Research Involving Human Subjects (ref. 2018-4110). All participants provided written consent before enrolment in the IBDREAM registry.

## Results

### Study population

**Fig. 1.**
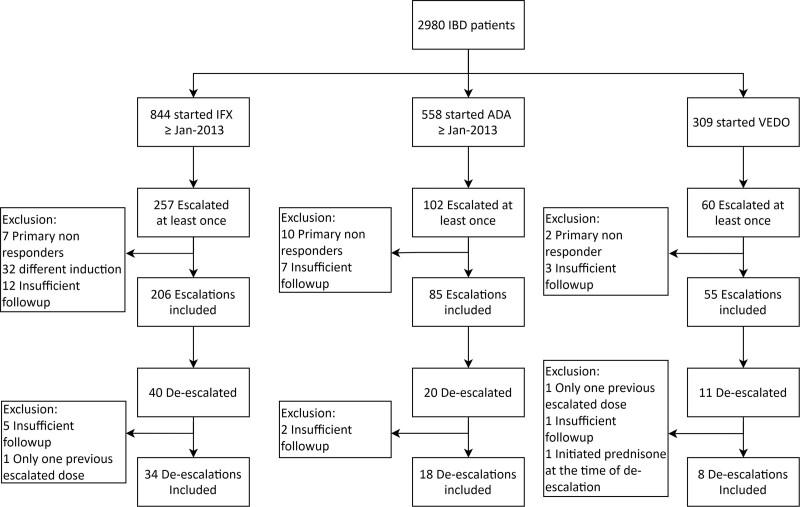
Flowchart de-escalation of biological therapy in inflammatory bowel disease patients. The total number of inflammatory bowel disease patients represents all patients included in the Dutch IBDREAM registry. The second step includes all patients that started the specific biological therapy after 1 January 2013. Data were extracted from the IBDREAM registry on 6 June 2020. ADA, adalimumab; IBD, inflammatory bowel disease; IFX, infliximab; VEDO, vedolizumab.

In total, 2980 IBD patients were enrolled in the IBDREAM registry. Among these patients, 844 started IFX treatment, 558 started ADA and 309 started VEDO after 1 January 2013. Therapy was escalated in 206 (24%) IFX users, 85 (15%) ADA users, and 55 (18%) VEDO users after a median of 12.9 months (IQR 6.4–25.1) for IFX, 7.5 months (IQR 3.9–16.1) for ADA, and 10.1 months (IQR 7.8–19.3) for VEDO. After at least one escalation, de-escalation was performed in 34 (17%) IFX users, 18 (21%) ADA users and 8 (15%) VEDO users (Fig. 1). An immunomodulator was used at the time of de-escalation in 59% for IFX, 39% for ADA and 38% for VEDO. At the time of de-escalation, one patient treated with IFX used 5 mg prednisone in the last week of tapering. Median follow-up after de-escalation comprised 18.1 months (IQR 9.7–30.7) for IFX, 31.8 months (IQR 10.7–39.4) for ADA and 15.9 months (IQR 9.8–24.0) for VEDO. Baseline characteristics are shown in Table 1.

**Table 1.** Baseline characteristics at the time of de-escalation

	Infliximab (*N* = 34)	Adalimumab (*N* = 18)	Vedolizumab (*N* = 8)
Sex, female, *N* (%)	20 (58.8)	9 (50.0)	5 (62.5)
Age (years), median (IQR)	37.4 (25.01–50.7)	33.6 (23.9–37.4)	47.7 (28.1–61.9)
BMI (kg/m^2^), median (IQR)	26.7 (23.1–31.2)	23.5 (21.7–25.0)	26.0 (22.6–32.2)
Age at IBD diagnosis (years), median (IQR)	28.0 (21.0–40.0)	25.5 (20.5–33.0)	33.0 (16.7–44.8)
Time between IBD diagnosis and de-escalation (years), median (IQR)	5.6 (1.8–9.4)	4.5 (2.6–6.8)	9.4 (3.5–23.9)
Time between biological initiation and first escalation (months), median (IQR)	7.0 (5.0–11.7)	11.1 (6.5–21.7)	9.2 (8.0–13.3)
Time between first escalation and first de-escalation (months), median (IQR)	9.7 (4.8–19.3)	5.7 (1.4–17.1)	8.0 (7.0–10.8)
IBD type, *N* (%)			
Crohn’s disease	20 (58.8)	15 (83.3)	5 (62.5)
Ulcerative colitis	13 (38.2)	3 (16.7)	2 (25.0)
IBD-U	1 (3.0)	0 (0)	1 (12.5)
Montreal CD			
Disease location, *N* (%)			
Ileum	4 (20.0)	4 (22.2)	1 (16.7)
Colon	5 (25.0)	4 (22.2)	1 (33.3)
Ileocolon	10 (50.0)	7 (46.7)	3 (50.0)
Upper GI involvement	2 (10.0)	2 (13.3)	1 (16.7)
Disease behavior, *N* (%)			
Stricturing	3 (15.0)	4 (26.7)	3 (50.0)
Penetrating	4 (20.0)	3 (20.0)	1 (16.7)
Perianal disease	4 (20.0)	3 (20.0)	1 (16.7)
Montreal UC/IBD-U, *N* (%)			
Proctitis	1 (7.1)	0 (0)	0 (0)
Left-sided	6 (42.9)	1 (33.3)	2 (66.7)
Pancolitis	7 (50.0)	2 (66.7)	1 (33.3)
Prior biological, *N* (%)			
None	29 (85.3)	15 (83.3)	0 (0)
Anti-TNF			
1	5 (14.7)	3 (16.7)	4 (50.0)
2	0 (0)	0 (0)	4 (50.0)
Vedolizumab	1 (2.9)	0 (0)	-
Ustekinumab	0 (0)	0 (0)	0 (0)
Concomitant medication, *N* (%)			
Mesalamine	8 (23.5)	3 (16.7)	2 (25.0)
Corticosteroids	1 (2.9)	0 (0)	0 (0)
Immunomodulator	20 (58.8)	7 (38.9)	3 (37.5)
Use of immunomodulator before de-escalation (years)	1.5 (1.1–2.4)	1.9 (1.0–3.6)	Range: 1.7–2.5
Prior intestinal resection, *N* (%)	4 (11.8)	4 (22.2)	3 (37.5)
Smoking status, *N* (%)			
Active smoker	7 (20.6)	6 (33.3)	2 (25.0)
Previous smoker	6 (17.6)	2 (11.1)	2 (25.0)
Never smoked	21 (61.8)	10 (55.6)	4 (50.0)

BMI, body mass index; CD, Crohn’s disease; IBD, inflammatory bowel disease; IBD-U, inflammatory bowel disease unclassified; IQR, interquartile range; TNF, tumour necrosis factor; UC, ulcerative colitis.

### De-escalation characteristics and outcomes per biological

Characteristics of the de-escalation are presented in Table 2.

**Table 2.** Outcome and characteristics of de-escalation

	Infliximab (*n* = 34)	Adalimumab (*n* = 18)	Vedolizumab (*n* = 8)
Time remained on preceding escalated dose (months), median (IQR)	7.5 (3.7–10.9)	5.7 (1.4–17.1)	7.8 (5.2–9.4)
Time remained on de-escalated regimen (months), median (IQR)	14.0 (6.3–19.4)	11.2 (6.5–37.2)	10.1 (3.2–21.4)
Escalations before de-escalation, *N* (%)			
1	21 (61.8)	18 (100)	6 (75.0)
2	11 (32.3)	0 (0)	2 (25.0)
3	2 (5.9)	0 (0)	0 (0)
De-escalation outcome, *N* (%)			
Successful	30 (88.2)	16 (88.9)	8 (100.0)
Unsuccessful	4 (11.8)	2 (11.1)	0 (0)
Type of de-escalation, *N* (%)			
Dose reduction	9 (26.5)	0 (0)	0 (0)
Increase dosing interval	24 (70.6)	18 (100)	8 (100)
Both dose reduction and increase dosing interval	1 (2.9)		
Dose reduction, *N* (%)	10 (29.4)	0 (0)	0 (0)
2.5 mg/kg	5 (50.0)	-	-
5 mg/kg	5 (14.7)	-	-
Increase in dosing interval, *N* (%)	25 (73.5)	18 (100)	8 (100)
1 week	5 (20.0)	18 (100)	3 (37.5)
2 weeks	19 (76.0)	0 (0)	5 (62.5)
3 weeks	0 (0)	0 (0)	0 (0)
4 weeks	1 (4.0)	0 (0)	0 (0)
Trough levels (µg/ml), median (IQR)	12.0 (7.9–16.0)	8.2 (7.3–11.8)	32.0 (15.6–41.0)
Available for, *N* (%)	23 (67.6)	6 (33.3)	5 (62.5)
Reason for unsuccessful de-escalations, *N* (%)	4 (11.8)	2 (11.1)	0 (0)
Insufficient response	4 (100)	2 (100)	-
Adverse event	0 (0)	0 (0)	-

IQR, interquartile range.

#### Infliximab

IFX was de-escalated in 34 patients at a median of 9.7 months (IQR 4.8–19.3) after the first escalation. Nearly 38% of these patients had received two or more escalations before the de-escalation. Patients used a stable escalated regimen for a median of 7.5 months (IQR 3.7–10.9) before de-escalation. Trough levels before de-escalation were available in 23 (68%) patients with a median of 12.0 µg/ml (IQR 7.9–16.0). Faecal calprotectin levels were available in 20 (59%) patients with a median level of 69 µg/g (IQR 28–254). Dosing interval was increased in 25 (74%) patients and was most often prolonged by 2 weeks (*n* = 19/25). After de-escalation, 12 (35%) patients used the IFX dose at 5 mg/kg every 8 weeks (Supplementary Table 1, Supplemental digital content 1, *http://links.lww.com/EJGH/A742*). De-escalation was successful in 30 (88%) patients and unsuccessful in 4 (12%) patients. After de-escalation, patients continued to use the de-escalated regimen for a median of 14.0 months (IQR 6.3–19.4). Information on follow-up after de-escalation is presented in Supplementary Figure 1, Supplemental digital content 1, *http://links.lww.com/EJGH/A742*.

#### Adalimumab

ADA was de-escalated in 18 IBD patients at a median of 5.7 months (IQR 1.4–17.1) after escalation. Trough levels before de-escalation were available in six (33%) patients with a median level of 8.2 µg/ml (IQR 7.3–11.8). Faecal calprotectin levels were available in nine (50%) patients with a median level of 135 µg/g (IQR 38–324). De-escalation was successful in 16 (89%) patients. After de-escalation, patients continued to use the de-escalated regimen for a median of 11.2 months (IQR 6.5–37.2). Information on follow-up after de-escalation is presented in Supplementary Figure 2, Supplemental digital content 1, *http://links.lww.com/EJGH/A742*.

#### Vedolizumab

VEDO was successfully de-escalated in all eight IBD patients at a median of 8.0 months (IQR 7.0–10.8) after the first escalation. Two (25%) patients had received more than one escalation before de-escalation. Patients used a stable escalated regimen for a median of 7.8 months (IQR 5.2–9.4) before de-escalation. Trough levels before de-escalation were available for five (63%) patients with median levels of 32.0 µg/ml (15.6–41.0). Faecal calprotectin levels were available in seven (88%) patients with a median level of 109 µg/g (IQR 21–200). The dosing interval was most often increased by 2 weeks (*n* = 5/8; 63%). After de-escalation, two (25%) patients used the standard VEDO maintenance dosing interval of 300 mg every 8 weeks. After de-escalation, patients continued to use the de-escalated regimen for a median 10.1 months (IQR 3.2–21.4). Information on follow-up after de-escalation is presented in Supplementary Figure 3, Supplemental digital content 1, *http://links.lww.com/EJGH/A742*.

### Use of diagnostic measures before de-escalation and outcomes of de-escalation

**Fig. 2.**
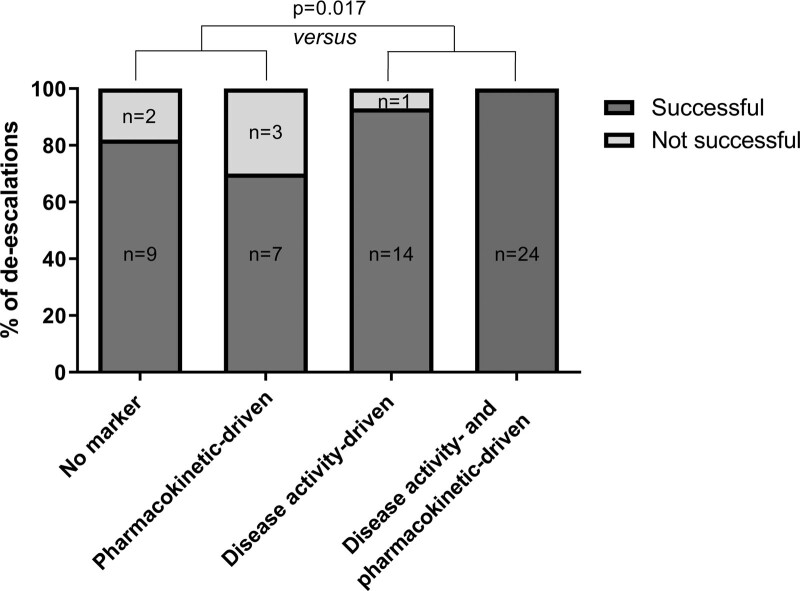
De-escalation outcomes stratified per diagnostic measure used before de-escalation. Pharmacokinetic-driven – de-escalation based on clinical symptoms and therapeutic or supratherapeutic trough levels. Disease activity-driven – de-escalation based on faecal calprotectin less than 200 µg/g, resolution of perianal fistula drainage or closure or endoscopic remission based on the absence of ulcers. Both – de-escalation based on a combination of pharmacokinetic-driven and disease activity-driven. No marker – de-escalation based on only clinical symptoms.

Before de-escalation, assessment of trough levels, faecal calprotectin or endoscopy was performed in 79% IFX patients, 67% ADA patients and 88% VEDO patients. More specifically, faecal calprotectin was measured before 57% of all de-escalations and endoscopic assessment was performed before 12% of all de-escalations. Of the 60 de-escalations, 10 (17%) were pharmacokinetic-driven, 15 (25%) were disease activity-driven, 24 (40%) were both pharmacokinetic- and disease activity-driven and 11 (18%) were no marker-driven. De-escalations were successful in 97% if remission was confirmed through objective diagnostic measures of disease either with or without the support of trough levels versus 76% if based on clinical symptoms only, either with or without the support of trough levels (*P* = 0.017) (Fig. 2).

### De-escalation stratified for time frames 2013–2016 versus 2017–2019

#### Infliximab

Twenty-seven (79%) patients were de-escalated between 2017 and 2019 versus seven (21%) patients in 2013–2016. The median time between first escalation and de-escalation was similar for these time frames [9.7 months (IQR 5.8–19.7) versus 7.2 months (IQR 3.2–22.5), respectively]. Trough levels were more often available in patients who de-escalated in 2017–2019 compared with 2013–2016 (75 versus 33%, respectively; *P* = 0.048).

#### Adalimumab

Twelve (66%) patients were de-escalated in the time frame 2017–2019 versus six (33%) patients between 2013 and 2016. In the time frame 2017–2019, the median time to escalation tended to be longer [17.4 months (IQR 5.7–24.5) versus 10.0 months (IQR 9.5–11.9); *P* = 0.303] whereas the time between escalation and de-escalation was similar [5.3 months (IQR 1.4–18.5) versus 6.1 months (IQR 4.2–15.6)]. Trough levels before de-escalation were available in 33% of patients in both time frames.

### Drug survival after de-escalation

**Fig. 3.**
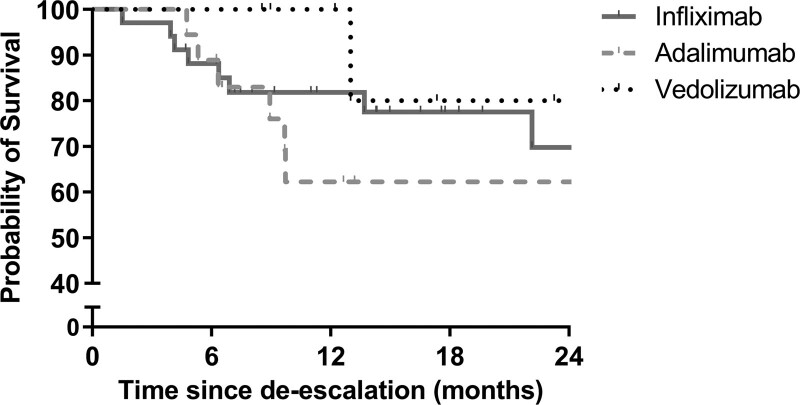
Kaplan–Meier survival curves demonstrating infliximab, adalimumab and vedolizumab persistence on de-escalated regimen. Median continued use of the de-escalated regimen or further de-escalation per biological therapy were as follows: infliximab 14.3 months (IQR 6.9–28.9), adalimumab 11.1 months (IQR 6.5–37.2) and vedolizumab 15.2 months (IQR 9.8–24.0). IQR, interquartile range.

The probability of remaining on the de-escalated regimen or further de-escalation after 12 months was 81.8% for IFX, 62.2% for ADA and 100% for VEDO. After 24 months, this probability was 69.8% for IFX, 62.2% for ADA and 80.0% for VEDO (Fig. 3).

### Re-escalation after de-escalation

Overall, nine (15%) patients were re-escalated due to an unsuccessful de-escalation or relapse at least 6 months after de-escalation. All patients were successfully re-escalated (Supplementary Figures 1–3, Supplemental digital content 1, *http://links.lww.com/EJGH/A742*).

## Discussion

In this study, we assessed the frequency and success rate of de-escalation of IFX, ADA or VEDO therapy in IBD patients who were previously escalated, and the extent to which physicians performed subjective and objective de-escalations. In this large retrospective multicentre cohort, approximately 20% of IBD patients on escalated biological therapy subsequently de-escalated. This was successful in almost all IBD patients. De-escalation strategies varied between interval increase by 1–4 weeks, dose reduction and a combination of interval increase and dose reduction. De-escalation driven by objective disease assessment was more often successful (97%) than pharmacokinetic-driven or no marker-driven de-escalations (76%).

In line with our results, high success rates of IFX de-escalation were reported in IBD patients in clinical and biochemical remission with relapse rates of 16% one year after de-escalation [13], similar to the average loss of response rate of IFX [21]. VEDO de-escalation success rate and drug survival were similar to a previous study that assessed de-escalation from 4- to 8-weekly in the extension study of the phase III studies (88% maintained 8-weekly dosing up to 56 weeks) [22]. The success rate of IFX and ADA de-escalation in our study (88–89%) was higher compared to other studies that used the same definition of de-escalation success (63–80%) [2,3]. However, these studies reflect clinical practice in which escalation and de-escalation were more often performed based on clinical symptoms rather than objective measures which are in line with the success rate of no marker-driven de-escalations reported in our study (76%). Moreover, the difference in success rate could be due to more strict or conservative physicians’ clinical decisions to de-escalate. Indeed, we observed a relatively low number of de-escalations in our cohort (16–21%) compared with previous studies that assessed de-escalation after prior escalation of adalimumab (54–71%) [2,3]. These high success rates of de-escalation among different real-world studies suggest that this strategy may be generalized to a larger subset of biological-treated IBD patients than we observed in this study.

De-escalations based on objective disease assessment with or without assessment of trough levels were statistically significantly more often successful compared with de-escalations based on an assessment of symptom-based clinical disease activity either with or without trough level measurement. Currently, no clear recommendations are available to guide the de-escalation of biological therapy. Clinicians may be reluctant to de-escalate after regaining therapeutic effect after dose escalation. Therefore, stepwise de-escalation may seem more reasonable than de-escalating back to the dose or interval that resulted in a loss of response. However, there are guidelines in place for biological discontinuation which may be applied to the de-escalation of biological therapy. These guidelines recommend achieving deep remission before discontinuation to reduce the relapse risk [23,24]. Yet this recommendation is not easily achieved in clinical practice because repeated endoscopic assessments are often not feasible and not always accepted by patients. Instead, biomarkers such as faecal calprotectin are used as a surrogate marker for luminal disease activity. These markers may be used to identify patients at risk to develop a disease relapse, even before manifesting symptoms. The use of biomarkers results in improved clinical and endoscopic outcomes when implemented in daily care to support clinical decision-making compared to symptom-driven decision-making [25]. Furthermore, supratherapeutic IFX and ADA trough levels before de-escalation are associated with a reduced relapse risk after de-escalation [13,18,26]. These findings underline the importance of the use of objective measures for therapeutic decision-making. In our study, we found that objective markers of disease activity were used in only 66% of patients before de-escalation. More specifically, in 57% faecal calprotectin was measured in whom 75% was <200 μg/g and thus demonstrating biochemical remission according to the recently revised STRIDE-II criteria [27]. When considering the previous STRIDE recommendations, endoscopic assessment was performed only in 12% and it demonstrated deep remission [28].

De-escalation of biological therapy may be considered in IBD patients who are in remission based on objective disease measures. Therapeutic or supratherapeutic trough levels may further support the decision to de-escalate. Our data showed that in carefully selected patients, this strategy has a high success rate based on the high percentage of patients continuing the de-escalated dose. In addition, re-escalation following disease recurrence was successful in the majority of patients. This may help physicians and patients to consider de-escalation after escalation with a ‘rescue’ strategy available.

Strengths of this multicentre study include the extensive follow-up of a large biological-treated IBD population. Stringent definitions of outcome measures and systematic data recordings after the introduction of the electronic healthcare records in 2013 ensured the quality of data and study outcomes. This is the first study that reports outcomes of differentiated de-escalation strategies. Limitations of our study include the relatively small number of patients that de-escalated in real life and therefore, statistical identification of possible predictors could not be performed. Moreover, de-escalations were not performed in a standardized manner including a wide range of changes in dose and intervals and more dose escalations were allowed before performing dose de-escalation. Therefore, dose de-escalation was subject to the physician’s and patient’s willingness to de-escalate. However, this reflects the daily practice and shows that shared decision-making is important to support the strategy of de-escalation. Considering the heterogeneity in dose de-escalation strategies and the relatively small number of patients, this study was mainly hypotheses generating. Future studies should prospectively assess the outcomes of standardized de-escalation. Lastly, endoscopic measures were only performed in 12% before de-escalation, and therefore data on mucosal healing or improvement were largely not available.

### Conclusion

In conclusion, de-escalation after biological dose escalation was successful in the majority of carefully selected IBD patients. Objective assessment of remission increased the likelihood of successful de-escalation. Biological de-escalation following prior dose escalation may be considered in case of objectively determined remission and therapeutic or supratherapeutic trough levels.

## Acknowledgements

None.

### Conflicts of interest

R.L.W. has participated in advisory boards, or as a speaker or consultant for the following companies: Abbvie and Janssen. J.M.J. has served on advisory boards, or as a speaker or consultant for Abbvie, Amgen, Ferring, Fresenius, Janssen, MSD, Pfizer and Takeda. T.E.H.R. has served as a speaker or consultant for Ferring, Janssen and Takeda. F.H. has served on advisory boards, or as a speaker or consultant for Abbvie, Celgene, Janssen Cilag, MSD, Takeda, Celltrion, Teva, Sandoz and Dr Falk, and has received unrestricted grants from Dr Falk, Janssen-Cilag and Abbvie. For the remaining authors, there are no conflicts of interest.

## Supplementary Material


