# Supplementary material for: De-escalation of biological therapy in inflammatory bowel disease patients following prior dose escalation
Source: Eur J Gastroenterol Hepatol. 2022 Mar 1;34(5):488–95. doi: 10.1097/MEG.0000000000002336 (PMC8983943; doi:10.1097/MEG.0000000000002336)
Supplement: Supplementary file 1 [file ejgh-34-488-s001.pdf]

**SUPPLEMENTARY FILES**

| **SUPPLEMENTARY TABLE 1** | | | | |
|---|---|---|---|---|
| | | **Infliximab (n=34)** | **Adalimumab (n=18)** | **Vedolizumab (n=8)** |
| **Dose after de-escalation** | | | | |
| 5.0 mg/kg | N (%) | 26 (76.5) | - | - |
| 7.5 mg/kg | N (%) | 5 (14.7) | - | - |
| 10 mg/kg | N (%) | 3 (8.8) | - | - |
| **Frequency after de-escalation** | | | | |
| 1x/ 4 weeks | N (%) | 1 (2.9) | - | 0 (0) |
| 1x/ 5 weeks | N (%) | 1 (2.9) | - | 2 (25.0) |
| 1x/ 6 weeks | N (%) | 13 (38.2) | - | 3 (37.5) |
| 1x/ 7 weeks | N (%) | 2 (5.9) | - | 1 (12.5) |
| 1x /8 weeks | N (%) | 17 (50.0) | - | 2 (25.0) |
| **Maintenance dose after de-escalation** | | | | |
| 5 mg/kg 1x/ 8 wk | N (%) | 12 (35.3) | - | - |
| 5 mg/kg 1x/ 7 wk | N (%) | 2 (5.9) | - | - |
| 5 mg/kg 1x/ 6 wk | N (%) | 10 (29.4) | - | - |
| 7.5 mg/kg 1x/ 8 wk | N (%) | 3 (8.8) | - | - |
| 5mg/kg 1x/ 5 wk or 7.5 mg/kg 1x/ 7 wk | N (%) | 1 (2.9) | - | - |
| 5mg/kg 1x/ 4 wk or 7.5 mg 1x/ 6 wk or 10mg/kg 1x/ 8 wk | N (%) | 5 (14.7) | - | - |
| 10 mg/kg 1x/ 7 wk | N (%) | 0 (0) | - | - |
| 10 mg/kg 1x/ 6 wk | N (%) | 1 (2.9) | - | - |

**SUPPLEMENTARY FIGURE 1.** *Flowchart Treatment changes and outcomes for inflammatory bowel disease patients using infliximab included for de-escalation analyses.*

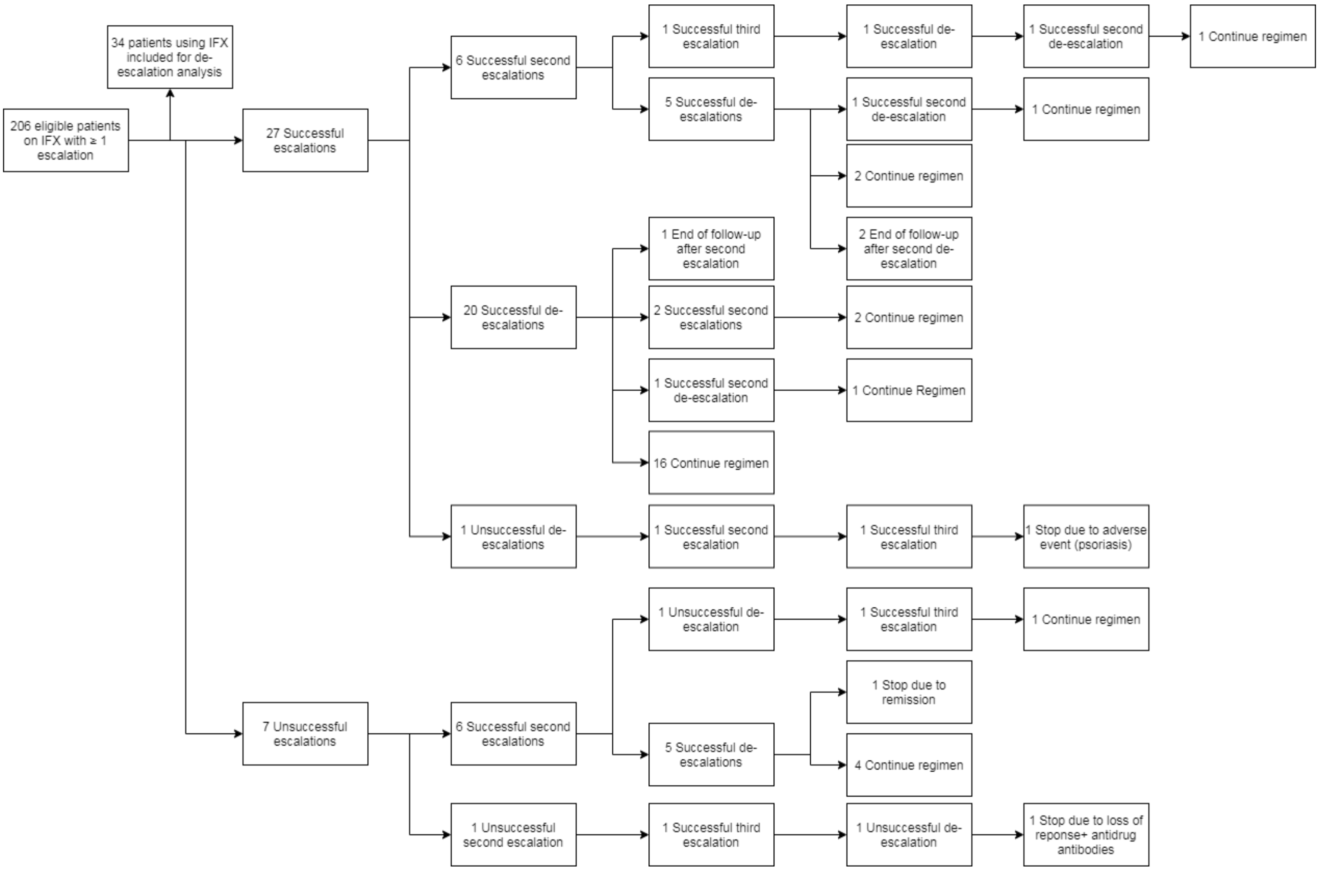


Four (12%) patients were re-escalated after either successful or unsuccessful de-escalation. All re-escalations were successful.

**SUPPLEMENTARY FIGURE 2.** *Flowchart Treatment changes and outcomes for inflammatory bowel disease patients using adalimumab included for de-escalation analyses.*

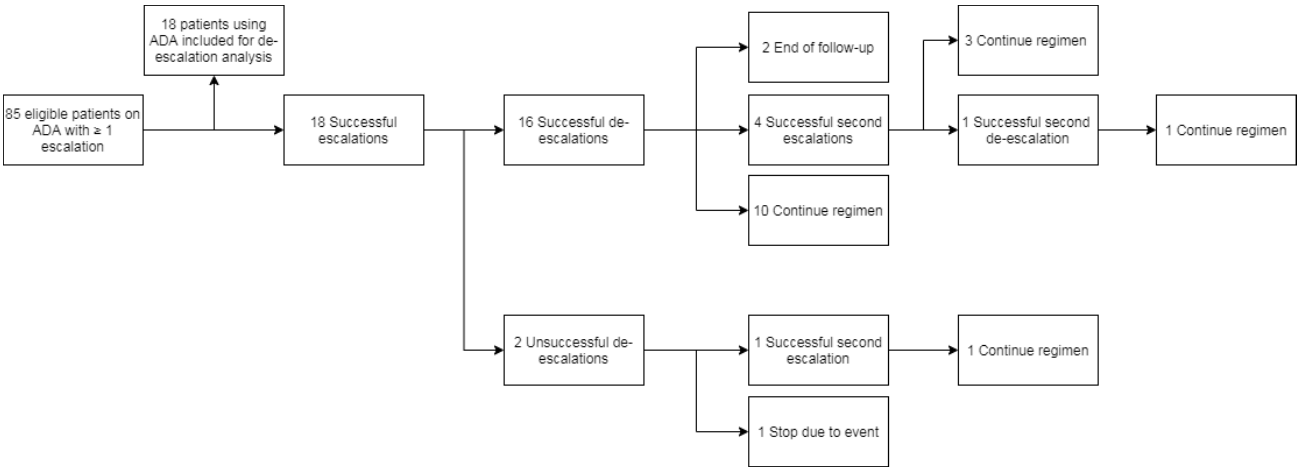


Seven (39%) patients were re-escalated after either a successful or unsuccessful de-escalation. Of these patients, five had sufficient follow-up and all were successfully re-escalated.

**SUPPLEMENTARY FIGURE 3.** *Flowchart Treatment changes and outcomes for inflammatory bowel disease patients using vedolizumab included for de-escalation analyses.*

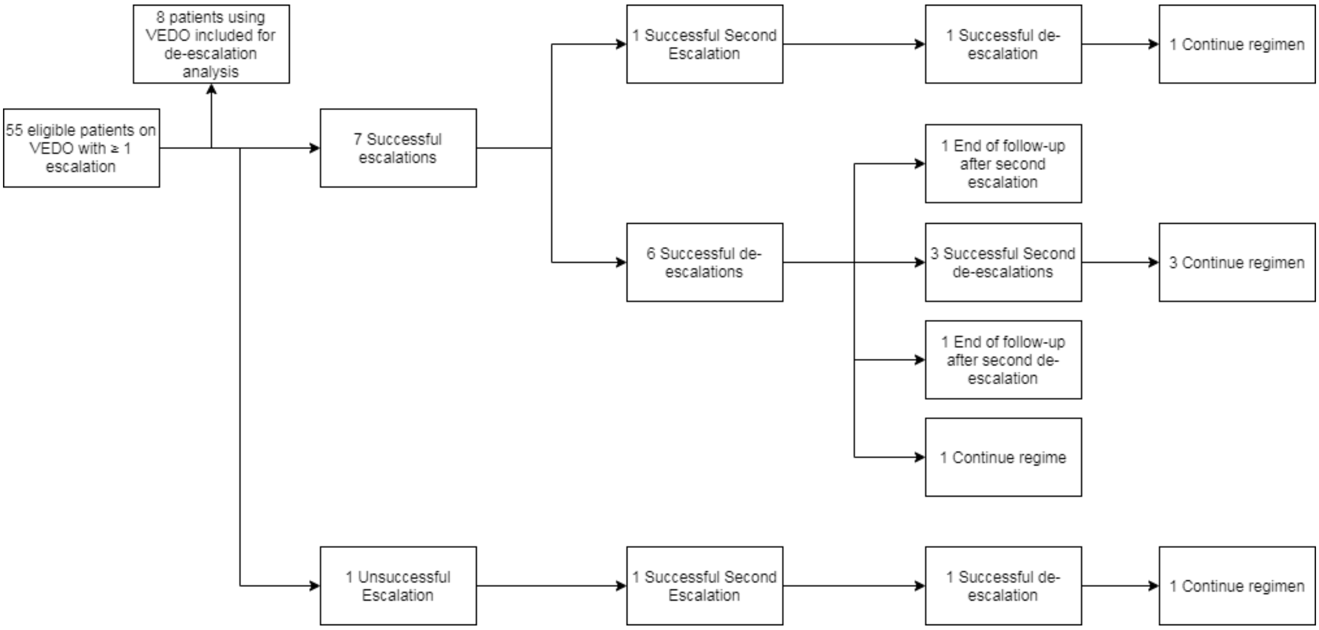


One (12.5%) patient was re-escalated after successful de-escalation. This patient had insufficient follow-up to determine the outcome of re-escalation.
